# Dietary diversity among Sundarbans forest-dependent communities: Prevalence, determinants, and livelihood implications

**DOI:** 10.1371/journal.pone.0342800

**Published:** 2026-02-11

**Authors:** Md. Tanvir Hossain, Tunvir Ahamed Shohel, Md. Nasif Ahsan, Md. Nazrul Islam

**Affiliations:** 1 Sociology Discipline, Social Science School, Khulna University, Khulna, Bangladesh; 2 Economics Discipline, Social Science School, Khulna University, Khulna, Bangladesh; 3 Forestry and Wood Technology Discipline, Life Science School, Khulna University, Khulna, Bangladesh; Yangtze University, MALAWI

## Abstract

The resources of the Sundarbans mangrove forest have provided livelihoods for communities in Bangladesh and India that depend on it. However, the role of the Sundarbans in ensuring the household dietary diversity of Sundarbans mangrove forest resource-dependent communities (SMFRDCs) remains unexplored. Considering the Sustainable Livelihood Approach, this cross-sectional survey study was conducted in three coastal districts (Khulna, Satkhira, and Bagerhat) to assess the prevalence and determinants of dietary diversity among SMFRDCs. Data were collected using a structured interview schedule from 782 households selected through a multistage stratified random sampling process over three months in 2023. Relevant statistical tests were conducted to assess the prevalence of dietary diversity and identify its determinants among households in the immediate vicinity of the Sundarbans. The one-sample binomial test showed that honey collectors and households in Shyamnagar *Upazila* had higher dietary diversity than those involved in other occupations and residing in other areas. The results of the binary logistic regression analysis indicated that individuals with higher education and those involved in multiple seasonal occupations were more likely to have diversified diets; however, spatial location had an inverse effect on the diets of SMFRDCs. Household assets, including domestic, transport, and livestock assets, as well as livelihood capitals such as social, natural, financial, and political, were positively associated with a diversified diet. In contrast, human and physical capital were negatively associated with household dietary diversity. The findings further show that physical vulnerability, along with household food insecurity, negatively affected dietary diversity among forest-proximate households. To ensure a sustainable, proper, and protein-enriched diet for forest-resource dependent people in the Sundarbans mangrove forest, context-specific, tailored, and well-integrated strategies are needed, including promoting alternative livelihoods, such as climate-smart agriculture, along with awareness regarding the consumption of locally available nutritious foods and government-aided food assistance programs, specifically during seasonal unemployment. Moreover, to improve access to diversified food items essential for the physical and mental development and well-being of forest-adjacent marginalized communities in coastal Bangladesh, certain factors suggested by the Sustainable Livelihood Approach, such as vulnerability, assets, policies and structures, and livelihood strategies, should be considered to ensure the sustainability of livelihood and resources, especially for climate-vulnerable communities.

## Introduction

The Sundarbans mangrove forest (SMF) – the largest single-track mangrove forest in the world [1] – is unique because it is composed of forest, coastal, and wetland ecosystems, housing both aquatic and terrestrial flora and fauna, which not only protects biodiversity but also has unique arrangements of natural species that provide livelihoods for animals as well as humans [1,2]. Because of its unique reserve of natural resources – both biotic and abiotic, the SMF has attracted people from varying ethnic and religious backgrounds, such as Munda, and of financial interests, including fishermen, *Nypa* leaf collectors, honey collectors, crabbers, and woodcutters, for centuries [3]. In fact, 7.5 million people living within and adjacent to the districts of the SMF extract natural products, including fish, crab, shrimp fry, honey, timber, leaves, and pulp, for their livelihoods, especially people living around the adjacent areas of the SMF, also known as the Sundarbans mangrove forest resource-dependent communities (SMFRDCs) [3–5].

Although there are numerous studies on the potential of Sundarbans as a source of livelihood for the SMFRDCs [5–7], their struggles, especially during crises such as COVID-19 [8,9] and natural disasters [10] remained almost in the shadow. Likewise, access to basic amenities, such as food or dietary diversity, one of the primary goals of the Sustainable Development Goals (SDGs), has not been investigated so far. Dietary diversity, also known as household dietary diversity, is an important indicator of a household’s ability to provide a diverse range of food for good nutrition for its members [11], and is often measured by the household dietary diversity score (HDDS) – a proxy measurement of a household’s economic ability to access a variety of food items over a given reference period [11,12]. Recent studies in Bangladesh have documented that the average HDDS varied from 6.16 in 2010 to 6.3 in 2016, and households in cities had better HDDS [13,14], while in rural areas, the HDDS was significantly lower than that of municipalities and other small municipality areas [13]. It is also evident that the HDDS score was significantly lower in the Khulna division than in the Chattogram, Dhaka, and Sylhet divisions [13,14]. Another study, documenting intra-household dietary diversity among agricultural farming households in Bangladesh, found that the individual dietary diversity score (IDDS) for men increased from 2.778 to 3.002 over six years, whereas the IDDS for women and children was lower than that of men [15]. Consequently, members of poorer families, particularly women and children, experience a deficiency of necessary micronutrients, resulting in malnutrition and other health comorbidities [16,17]. A recent study on forest resource-dependent communities in the Sundarbans disclosed that the HDDS among the Sundarbans-proximate households was 4.8 [18], lower than the national average of 6.3 [14] and other occupational groups in Bangladesh [19,20], while the plant- and animal-based dietary scores were only 2.8 and 2, respectively [18].

There is no denying that assuring dietary diversity and reducing food insecurity for a natural hazards-prone region such as southwestern coastal Bangladesh with a marginalized population, that is, SMFRDCs, is important to ensure not only quality food intake but also a reduction in non-communicable diseases [13,14,20,21], especially during crisis moments [22]. Studies documented that forest resource-dependent communities in the Sundarbans frequently experienced reduced food consumption [8,9,23] due to seasonal unemployment, government-imposed bans, and natural disasters, which reduced household income and affected households’ capacity to access protein-rich foods [10,23]. Hence, addressing dietary diversity among Sundarbans adjacent communities requires context-specific empirical investigation to understand the dynamics of linkages among diverse sociodemographic, economic, environmental, and politico-cultural issues to revise and implement development policies to reduce hunger, malnutrition, and poverty, and to pursue the Sustainable Development Goals (SDGs), more specifically SDG 1 (no poverty), SDG 2 (zero hunger), SDG [13] (climate action), and SDG 15 (life on land), in order to ensure sustainable harvesting of forest resources and promoting stable income opportunities while protecting biodiversity. The SMF has long served as a source of livelihood for millions of SMFRDCs [6,7,24]. However, the role of forest dependency in livelihood outcomes, such as dietary diversity, has remained unexplored. Therefore, this study was designed to assess the prevalence of dietary diversity among SMFRDCs and identify factors associated with devising policies and strategies to ensure not only dietary diversity and reduction of food insecurity but also proper physical growth among residents living close to the SMF. It is needless to say, in the last few years, studies regarding dietary diversity have been carried out in different regions [14] on different sociodemographic and occupational groups [19,25–28] and different circumstances [11]; however, only a few, as per the best knowledge of the authors, targeted the SMFRDCs [18], whereas region-specific, occupational group-targeted interventions and policies are needed to ensure sustainable and diversified diets for these marginalized people. This study, based on representative populations from three coastal districts adjacent to the SMF, which are spatially, socio-culturally, and economically diverse, integrated the Sustainable Livelihood Approach (SLA) to assess the prevalence and to identify the factors that determine dietary diversity using rigorous statistical analysis.

## Theoretical and conceptual framework

The sustainable livelihood approach (SLA) was developed to understand and analyze the livelihoods of people experiencing poverty in existing social, institutional, and organizational vulnerability contexts [29]. SLA, in particular, sketches out critical issues and factors, both at the macro and micro levels, that affect livelihoods, particularly rural people, and draws attention to core influences, processes, and interactions – whether social, economic, environmental or political – which are highly dynamic and intersect from multidisciplinary perspectives, including natural and social sciences [29,30]. It sees livelihood as made up of the skills, resources, and activities of individuals that enable them to withstand shocks and strains – whether natural or artificial – and allow them to recover again to protect or improve existing assets and capabilities without endangering natural resources and the environment [31]. According to the SLA, the livelihood outcomes of individuals are influenced mainly by the *vulnerability context*, *livelihood assets*, *transforming structures and processes*, and *livelihood strategies* [29,30,32,33]. Integrating all four components, the SLA, as Scoones (32) elaborated, helps researchers and policymakers alike to understand how a particular context, with a combination of livelihood resources and strategies, under an institutional framework enables the marginalized people to achieve specific livelihood goals or outcomes.

Among these elements, devised by the Department for International Development (29), the *vulnerability context* reflects the external environment that directly influences not only an individual’s access to assets and alternative livelihood options but also their ability to cope with and recover from stresses associated with sustaining their livelihood [29,32]. *Livelihood assets* or *capital*, on the other hand, are critical for livelihoods, as they signify access to and use of tangible and intangible assets in their possession – whether material or social, and determine livelihood options for individuals within their communities [29,32]. *Human* capital refers to the skills, knowledge, physical capability, and overall health of individuals that enable them to pursue different livelihood strategies successfully [29,32,34]. *Social* capital entails social resources in the form of relationships, networks, affiliations, and associations among individuals, and membership in formal or informal groups, that are required to determine access to social resources to pursue livelihood options through coordinated actions [29,32,35]. *Natural* capital includes both natural resources (stocks of resources and season) and environmental services (infrastructure) in a particular setting, to determine livelihood opportunities and the production of goods using resource flows and useful services [29,32,34,35]. *Physical* capital refers to essential private and public infrastructures, including water supply, sanitation, technology, access to information, tools, and equipment, necessary to support people in meeting their basic necessities and enable them to be more productive by safeguarding access to tools and equipment to sustain their livelihoods [29,34–36]. *Financial* capital indicates to the capital base, such as cash, credit/debt, savings, and economic assets and technologies, that are used to achieve livelihood objectives, and it determines people’s ability to adopt different livelihood opportunities [29,32,34,35]. The Department for International Development (29) did not distinguish between *political* and *social* capital; it, however, insisted that political capital – the affiliation or association with political institutions or individuals – plays a critical role in accessing resources beyond the community [29]. Because livelihood strategies and outcomes among marginalized people are subject to local, regional, and global power, politics, and interests that may secure or endanger their rights, access, and governance issues [30,33]. *Transforming structures and processes* refers to the institutional influence on equitable access to livelihood resources and on strategies to cope with and enhance livelihood opportunities, to maximize livelihood outputs through competitive markets [29,32]. *Livelihood strategies* indicate the choices people make that not only provide flexibility in occupational opportunities but also enhance their capacity to withstand the shocks and stresses posed by situational circumstances [29,32]. *Livelihood outcomes* generally encompass a wide range of outputs that may be achieved through different livelihood strategies, including higher income, improved food security, and sustainable resource use [29,32].

**Fig 1:**
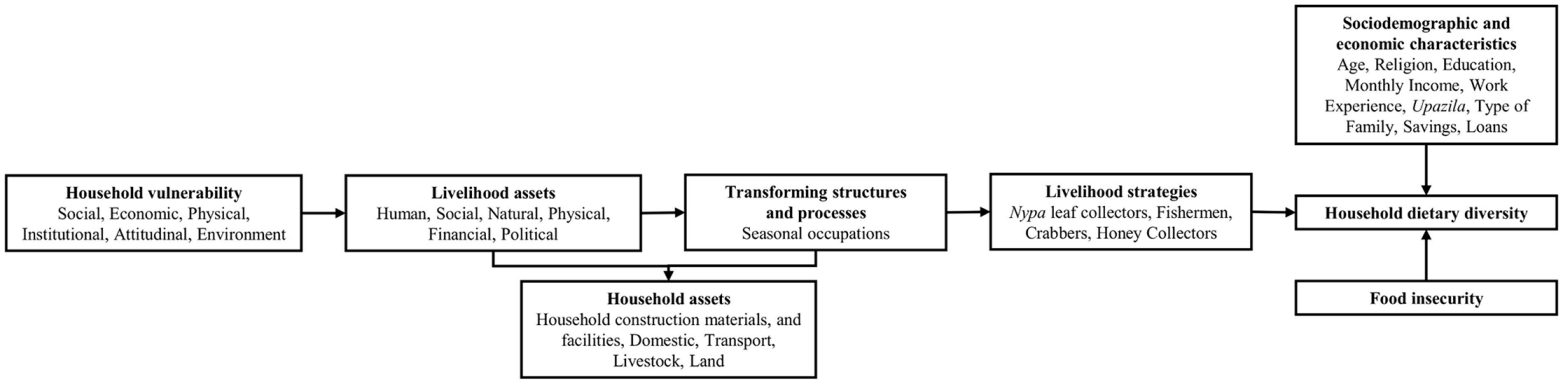
Conceptual framework integrating the sustainable livelihood approach in explaining dietary diversity among SMFRDCs.

In this study, the SLA framework was integrated to identify the determinants of dietary diversity among communities, depending particularly on the forest resources of the Sundarbans. Studies across the world have shown that the dietary diversity of forest resource-dependent marginalized communities was significantly determined by factors such as vulnerability, assets, policies, livelihood strategies, sociodemographic and economic characteristics, and the ability to secure food [37–40]. Likewise, the dietary diversity of SMFRDCs in the SMF is perceived to be determined by a range of factors such as household vulnerability, livelihood assets, household assets, transforming structures and processes, and livelihood strategies, alongside sociodemographic and economic characteristics and food insecurity (see Fig 1). A recent study explored the patterns of dietary diversity among forest-proximate people in Sundarbans, and they found that the HDDS among SMFRDCs, including plant- and animal-based diets, was lower than the national average and attributed this to differences in household and livelihood assets and vulnerabilities, along with sociodemographic and economic factors, as well as food insecurity [18]. Although they measured both plant- and animal-based diets among SMFRDCs, considering the SLA framework [18], they did not outline the extent or prevalence of dietary diversity across occupational groups and spatial locations of SMFRDCs, which would enable policymakers to design or implement region-specific, occupational-group-targeted interventions and policies to ensure sustainable, diversified diets for these spatially, socio-culturally, and economically diverse marginalized people.

## Materials and methods

### Study settings

This study was conducted in three southwestern coastal districts located in the low Ganges tidal floodplains and cyclone-prone zones of Bangladesh: Khulna, Satkhira, and Bagerhat [41]. Bangladesh has 64 districts, split into eight broad areas or divisions: the SMF – the largest mangrove forest in the world – lies in two of these divisions: Khulna and Barishal, in which Khulna (907 Km^2^), Satkhira (1632 Km^2^), and Bagerhat (1913 Km^2^) districts of the Khulna division have the largest share of the SMF [41–44]. These districts lie between N 21°36´ - N 22°54´ and E 88°54´ - E 89°20´ (Satkhira), N 21°41´ - N 23°00´ and E 89°14´ - E 89°45´ (Khulna), and N 21°49´ - N 22°59´ and E 89°32´ - E 89°98´ (Bagerhat) [42–44], covering the largest share of coastal zones in Bangladesh [45]. It is important to note that Khulna, Satkhira, and Bagerhat districts were selected as study areas under certain circumstances. For example, being close to the Bay of Bengal, Khulna, Satkhira, and Bagerhat districts are prone to frequent natural hazards, such as sea-level rise, cyclones, salinity intrusion, storm surges, floods, and shoreline erosion [46,47], affecting crop production [48] and the buying capacity of food items owing to the scarcity of necessary foods and employment opportunities, especially during crisis periods [8,9,49]. It is well documented that the overall dietary diversity in the southwestern coastal districts is lower than the national average [13,14], especially in Khulna, Satkhira, and Bagerhat districts [50].

### Participants and sampling strategy

**Fig 2:**
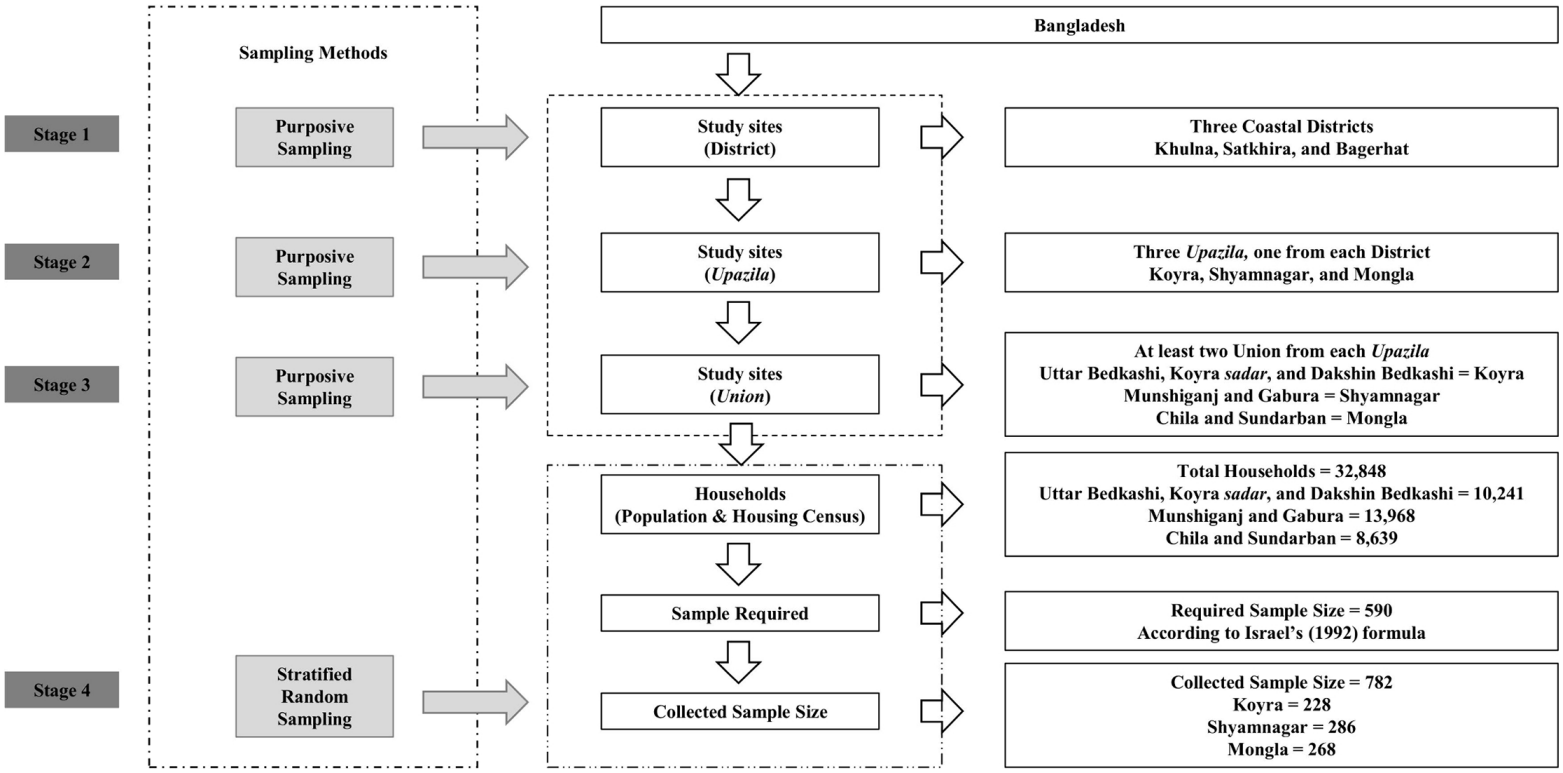
Sampling procedure.

In this study, Sundarbans-proximate households were selected based on certain predetermined characteristics: (i) they must be the male head of the household; (ii) they must be older than 15 years of age, especially in case where father was deceased or killed by Tiger or Crocodile attack; (iii) they must be dependent on the forest resources of the SMF for livelihood; and (iv) they must be residents of selected study areas of southwestern coastal districts. Considering the aforementioned specifications, the participants were selected using a multi-stage stratified random sampling approach [10,51,52]. In phase one, three southwestern coastal districts of Bangladesh – Khulna, Satkhira, and Bagerhat – were selected purposively. In phase two, three *Upazila* (sub-district) – one from each of the selected districts – were chosen purposively based on the severity and frequency of natural disasters [10,46,47], as well as the concentration of the SMFRDCs and proximity to the SMF [53,54]. In phase three, a minimum of two unions were purposively selected from each *Upazila*. Using a stratified random sampling technique, data were gathered from 782 individuals in the final phase based on occupational variety (see Fig 2) [55]. It is noteworthy that the required sample size for a population of 32,848 was 590 at a 95% confidence level with a 4% margin of error [56]. Notably, additional samples, for example, 192, were collected to cover more spatial locations and to obtain more responses from SMFRDCs to generalize the findings for a geographic and culturally diverse population with representative samples [55].

### Ethical issues

The institutional ethical clearance committee approved the study and granted ethical permission under reference number KUECC – 2023-07-42, dated 26 July 2023. At the beginning of the study, the participants, including underage household heads with their guardians’ assent (i.e., the mother), were provided with an overview of the objectives, and data collection was conducted with both verbal and written consent. The participants were assured that their information would be kept confidential and anonymous. To acknowledge their contributions, each participant was compensated for their time after the interview, as the interviews lasted 40–45 minutes. The participants had the right to decline participation without providing a reason in advance, and they were also given a designated period after the interviews to retract their responses if they wished.

### Procedures of data collection

For this cross-sectional survey, a structured interview schedule (SIS) – a subtype of the interview – was used to collect data at a single point in time in a face-to-face situation by setting predetermined, understandable, and meaningful question items where individuals share their experiences, thoughts, attitudes, opinions, and behaviors about a topic of mutual interest [55,57,58] from the perspective of the interviewee [59]. Compared to other sub-types of interviews, SIS facilitates the gathering of confidential and authentic information by fostering a friendly and secure rapport between interviewers and interviewees, where interviewers, without providing judgmental opinions, can clarify any uncertainties for interviewees [59]. In fact, interviewers using SIS can encourage deeper insights and verify the information provided by observing the interviewees’ gestures and body language [58–60]. The SIS in this study was developed after review of relevant literature and it contained several mutually exclusive sub-sections, focusing on socio-demographic information, household materials and facilities, livelihood assets, household vulnerability, dietary diversity, and household food security. After developing the SIS, the researchers trained 10 data enumerators – graduate and undergraduate students from a public university – for a week on the SIS’s content, with an emphasis on ethical issues to ensure uniformity in field data collection. The data enumerators were trained through role-playing to understand the process of rapport-building, and they were sent to the study areas for field trips to familiarize themselves with the life, livelihood, attitudes, and behaviors of SMFRDCs through interactions to develop trustworthiness [59]. Subsequently, the data enumerators pre-tested the SIS over 30 households of SMFRDCs with the exact specifications mentioned above for the participants, 10 from each selected district, to identify poorly worded and ambiguous questions and to ensure access to reliable and valid responses from the participants to reduce inconsistency and invalid information [55,61,62]. Following the pre-test of SIS, the researchers further edited it, and the data enumerators were deployed for fieldwork, which began in mid-August 2023 and ended in October 2023. It is important to note that the data were collected in the home settings of SMFRDCs at their convenience, without disrupting their daily activities, including household chores [10]. The SMFRDCs responded in Bangla – their native language – to reduce ambiguity in the ‘question and response’ session and to obtain clear statements from the participants. Each interview lasted for over 35 minutes without any break, and the data enumerators recorded the responses in a ‘pen and pencil’ approach on the printed SIS. If the head of a randomized household was absent, the data enumerators approached the head of the immediate next household [10].

### Measures

#### Socio-demographic and economic characteristics.

In this study, the socio-demographic and economic characteristics of SMFRDCs were measured by age (≤30, 31–45, 46 ≥), religion (Islam, Hindu), education (Not literate, Primary (Class I – Class V, Secondary and above [Class VI ≥]), monthly income in BDT (≤ 12,000, 12,001 ≥), work experience (≤ 15, 16–30, 31 ≥), *Upazila* (Shyamnagar, Koyra, Mongla), family type (Nuclear, Extended), savings (No, Yes), and loans (No, Yes).

#### Livelihood strategies.

In this study, the livelihood strategies of households proximate to the Sundarbans were assessed through four occupations: *Nypa* leaf collectors, fishermen, crabbers, and honey collectors, all of whom rely on the Sundarbans’ forest resources to sustain their livelihoods.

#### Transforming structures and processes.

In this study, seasonal occupations of SMFRDCs, measured in number, were used as an indicator of transforming structures and processes as forest resource-dependent households often experience seasonal unemployment either due to a ban imposed on the forest resource extraction by the Forest Department to ensure sustainable resource utilization, or due to a short window of resources, such as honey, to collect from the Sundarbans.

#### Household assets.

In this study, five distinct indices of household assets were developed, drawing insights from the relevant literature [63–65], to examine their association with the HDDS of SMFRDCs. The indices include ‘construction materials and facilities,’ ‘domestic assets,’ ‘transport assets,’ ‘livestock assets,’ and ‘land assets’; these indices contained four to seven items. To develop the composite score for each index, the scores assigned for each indicator were added together in order to develop the index total based on Equation i [66–68]:


$$\sum {\text{V}}_{\text{1}}+{\text{V}}_{\text{2}}+\;\;.\;.\;.\;.\;.\;+\;{\text{V}}_{\text{n}}$$ (i)

In this equation, *V*_1_ to *V*_n_ correspond to the scores assigned to the indicators within the index [66–68]. The scores assigned to each indicator were added together based on the equation (Equation 1) mentioned above. After calculating the index total, it was categorized into three equal classes based on percentile analysis to ensure a comprehensive assessment: “0” to “33” percentile denoted “Low”, “34” to “66” denoted “Medium”, and “67” or higher denoted “High”.

#### Livelihood assets.

In this study, indices comprising six livelihood capitals (i.e., human, social, natural, physical, financial, and political) were developed based on the sustainable livelihood approach and a review of the relevant literature [29,34,35,69–75]. It is essential to recognize that the Department for International Development (29) proposed five key livelihood capitals, identifying political capital as a component of social capital. Other studies have emphasized the need to explore the interplay between political capital and livelihood sustainability, particularly in regions susceptible to natural disasters [74,75]. Hence, political capital, along with other livelihood capital, was measured in this study using 58 items, where each index contained eight to 12 indicators, and the score assigned for each indicator was added to develop a composite score for each index based on the equation (Equation i) provided above [66–68]. After estimating the index total for each capital, the score was divided equally into three categories based on percentile analysis: “0” to “33” denoted “Low,” “34” to “66” denoted “Medium,” and “67” or higher denoted “High”.

#### Household vulnerability.

To measure household vulnerability, the household vulnerability assessment (HVA) scale, proposed by Rahman, Arif (66)was adopted and modified after drawing insights from other relevant literature [67,68,76]. HVA originally contained 43 items, split into ‘social vulnerability’ with nine items, ‘economic vulnerability’ with eight items, ‘physical vulnerability’ with ten items, ‘institutional vulnerability’ with eight items, ‘attitudinal vulnerability’ with five items, and ‘economic vulnerability’ with three items [66]. However, the modified HVA contained 56 items, divided into ‘social vulnerability’ with nine items, ‘economic vulnerability’ with eight items, ‘physical vulnerability’ with twelve items, ‘institutional vulnerability’ with fourteen items, ‘attitudinal vulnerability’ with nine items, and ‘economic vulnerability’ with four items. The score assigned to each indicator was added to develop a composite score for each index based on the equation (Equation i) provided above [66–68]. Upon calculating the score, the index total was strategically divided into three equal categories, leveraging percentile analysis to ensure a comprehensive assessment.: “0” to “33” percentile denoted “Low,” “34” to “66” denoted “Medium,” and “67” or higher denoted “High” [66–68].

#### Household food insecurity.

In this study, the household food insecurity was measured using the ‘Household Food Insecurity Access Scale (HFIAS) for measurement of food access’ [77]. HFIAS was developed to assess households’ degree of food insecurity over the past 30 days. HFIAS score is measured by two types of related questions: nine two-point Thurstone scale ‘occurrence question items’ – ‘0 = No’ and ‘1 = Yes’, followed by nine three-point Likert scale ‘frequency-of-occurrence’ question items – ‘1 = Rarely,’ ‘2 = Sometimes,’ and ‘3 = Often’ [77]. To estimate the composite score, for a negative response – ‘No’ – ‘0’ is assigned, while for ‘frequency-of-occurrence’ question items, the responses were retained; thus, the minimum score could be ‘0,’ reflecting ‘no food insecurity,’ whereas the maximum score could be ’27,’ suggesting the highest food insecurity [78]. In this study, the average of HFIAS was 9.9 (SD ± 3.68), and it was categorized as ‘1 = Food secure,’ ‘2 = Mildly food insecure access,’ ‘3 = Moderately food insecure access,’ and ‘4 = Severely food insecure access’ [77]. In this study, the internal consistency of the HFIAS was Cronbach’s α (alpha) = 0.788.

#### Household dietary diversity.

This study measured household dietary diversity using the ‘household dietary diversity score (HDDS) for household food access’ [12]. HDDS was developed to measure food consumption patterns at the intra-household level during a specific time period in a relatively shorter time, i.e., the last 24 hours [12]. It contained questions on 12 food groups, including cereals, root and tubers, vegetables, fruits, meat, poultry, and offal, eggs, fish and seafood, pulses, legumes, and nuts, milk and milk products, oil or fat, sugar or honey, and miscellaneous using a two-point Thurstone scale – ‘Yes’ and ‘No’ – and the value ranges from ‘0’ to ’12,’ where the higher value reflected greater dietary diversity at households [12]. The HDDS was calculated based on Equation (ii) [79,80], where an affirmative response, i.e., ‘Yes,’ was coded 1, while 0 was given for negative, e.g., ‘No.’


HDDS (0−12)=∑(A+B+C+D+E+F+G+H+I+J+K+L) (ii)

In this study, the average HDDS among SMFRDCs was 4.8 (SD ± 1.81), and the median was 5. Therefore, the median split was used to categorize the composite score [10,81] as either “No or Low dietary diversity = 0” (participants at or below the median score, i.e., 5 [≤ 5]) or “Medium or High dietary diversity = 1” (participants above the median score of 5 [6 ≥]). The internal consistency of HDDS, in this study, was Cronbach’s α (alpha) = ≤ 0.700.

### Analysis

In this study, data were analyzed, using IBM SPSS Statistics version 27 for Windows, into three consecutive phases: first, to assess the prevalence of dietary diversity among occupational groups and regions, an one-sample binomial test with a 95% confidence interval (CI) was performed; second, to measure the association between explanatory variables and outcome variable, the Pearson’s Chi-square (χ^2^) test for independence was performed at a 10% level of significance [59,82,83]. In the case of a 2 by 2 table, Yates’s Correction for Continuity (χ^2^_Yates_) was reported [84,85]. In addition, Cramer’s V (*φ*_c_) and Phi (*φ*) were reported to reflect the strength of the relationship in bivariate analysis for χ^2^ and χ^2^_Yates_, respectively [62,84,85]. The variables that were found to be statistically significant in the χ^2^ and χ^2^_Yates_ tests at a 10% level of significance, were retained in the multivariable binary logistic regression to determine whether the variables are affecting dietary diversity of the SMFRDCs or not by classifying the outcome variable into two categories, i.e., “no or low dietary diversity (≤ 5) = 0” and “moderate or high dietary diversity (6 ≥) = 1”, which would clarify the way in which predicting variables are affecting the outcome variable. In this study, the model adequacy of the binary logistic regression was assessed using pseudo *R*^2^ (Cox & Snell *R*^2^ and Nagelkerke R^2^ statistic) [84] and the Hosmer-Lemeshow test for ‘goodness of fit’ [86]. In this study, the Hosmer-Lemeshow test for ‘goodness of fit’ showed a *p* value of 0.271 (χ2 = 10.746), indicating a good model fit [84]. In addition, multicollinearity among the de*p*endent and predicting variables was assessed using the variance inflation factor (VIF) by running a multiple linear regression with the same outcome and determinants [85,86], accounting for the binary logistic regression’s sensitivity to high correlations among predicting variables [84]. The VIF assessment indicated that the mean VIF was below 2, indicating no multicollinearity among the predictors in this study [85]. The findings of the binary logistic regression were presented as unadjusted odds ratios (UORs) and adjusted odds ratios (AORs) with 95% CIs.

## Results

### Prevalence of household dietary diversity among SMFRDCs

**Fig 3:**
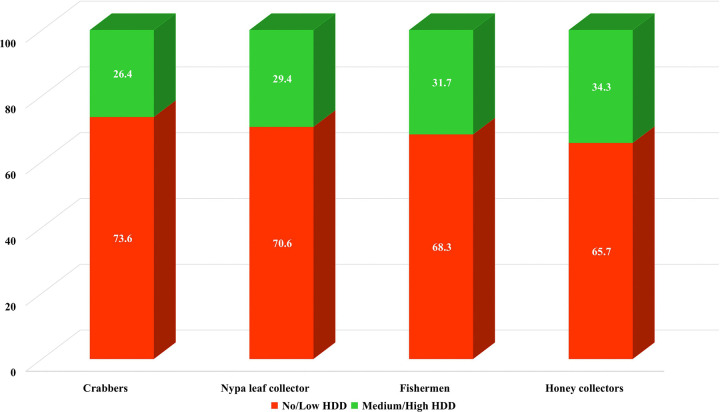
Prevalence of household dietary diversity by occupation among SMFRDCs.

**Fig 4:**
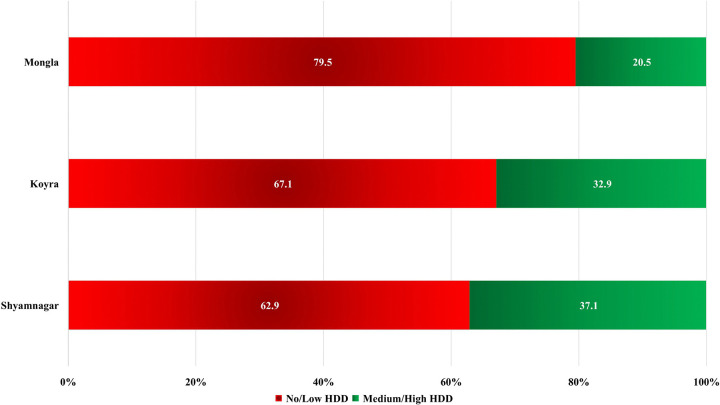
Prevalence of household dietary diversity by Upazila among SMFRDCs.

Fig 3 shows the prevalence of dietary diversity among the SMFRDCs by occupation. It is apparent that honey collectors (34.3%; 95% CI: 26.9%−42.3%), followed by fishermen (31.7%; 95% CI: 26.4%−37.3%), enjoyed relatively higher dietary diversity among the SMFRDCs, while crabbers (26.4%; 95% CI: 21.2%−32.3%) had the lowest dietary diversity. Fig 4 shows the prevalence of dietary diversity among the SMFRDCs by *Upazila*. The prevalence of dietary diversity was higher among SMFRDCs in Shyamnagar *Upazila* (37.1%; 95% CI: 31.6%−42.8%), followed by Koyra *Upazila* (32.9%; 95% CI: 27.0%−39.2%), while SMFRDCs of Mongla *Upazila* (20.5%; 95% CI: 16.0%−25.7%) had the least dietary diversity among all the spatial locations.

### Associated factors of household dietary diversity among SMFRDCs

Table 1 shows the overall prevalence of dietary diversity among the SMFRDCs and its associated factors. The findings indicated that only 30.2% (95% CI: 27.0%−33.5%) of the SMFRDC households had medium to high dietary diversity, while the remaining 69.8% (95% CI: 66.5%−73.0%) had no or low dietary diversity.

**Table 1:** Prevalence and associated factors of dietary diversity among SMFRDCs.

Variables	Household dietary diversity (%)		Test	P value	Effect size	P value
	No/Low dietary diversity (≤ 5)	Medium/High dietary diversity (6 ≥)				
**Prevalence**	**69.8%**	**30.2%**				
***Socio-demographic and economic characteristics***						
Age						
≤ 30	16.5	15.3	0.605^a^	0.739	0.028^c^	0.739
31-45	51.1	49.6				
46 ≥	32.4	35.2				
Religion						
Islam	94.7	90.7	4.348^b^	0.054	0.075^d^	0.037
Hindu	5.3	9.3				
Education						
Not literate	45.1	38.1	21.945^a^	< 0.001	0.168^c^	< 0.001
Primary (Class I – Class V)	39.7	32.2				
Secondary or higher (Class VI ≥)	15.2	29.7				
Monthly income (in BDT)						
≤ 12,000	55.7	52.5	0.533^b^	0.465	0.029^d^	0.419
12,001 ≥	44.3	47.5				
Work experience (in Year)						
≤ 30	24.0	26.7	1.226^a^	0.542	0.040^c^	0.542
31-45	46.5	42.4				
46 ≥	29.5	30.9				
*Upazila*						
Shyamnagar	33.0	44.9	19.090^a^	< 0.001	0.156^c^	< 0.001
Koyra	28.0	31.8				
Mongla	39.0	23.3				
Type of family						
Nuclear	68.5	55.5	11.593^b^	< 0.001	0.150^d^	< 0.001
Extended	31.5	44.5				
Savings						
No	56.2	39.8	17.081^b^	< 0.001	0.151^d^	< 0.001
Yes	43.8	60.2				
Loans						
No	36.1	24.2	10.153^b^	< 0.001	0.134^d^	0.001
Yes	63.9	75.8				
***Livelihood strategies***						
Occupation			3.054^a^	0.383	0.062^c^	0.383
*Nypa* leaf collector	15.4	14.8				
Fishermen	34.8	37.3				
Crabbers	32.6	27.1				
Honey collectors	17.2	20.8				
***Transforming structures and processes***						
Seasonal occupations			7.781^a^	0.051	0.100^c^	0.051
One	34.8	40.3				
Two	47.8	38.1				
Three	14.5	19.5				
Four	2.9	2.1				
***Household assets***						
Household construction materials and facilities						
Low (≤ 18)	43.0	35.2	10.973^a^	0.004	0.118^c^	0.004
Medium (19–20)	34.6	31.4				
High (21 ≥)	22.3	33.5				
Domestic assets						
Low (≤ 4,500)	38.3	23.7	15.919^a^	< 0.001	0.143^c^	< 0.001
Medium (4,501−11,900)	30.8	36.0				
High (11,901 ≥)	31.0	40.3				
Transport assets						
Low (≤ 10,000)	39.9	25.4	16.120^a^	< 0.001	0.144^c^	< 0.001
Medium (10,001–20,000)	40.1	46.6				
High (20,001 ≥)	20.0	28.0				
Livestock assets						
Low (≤ 3,000)	73.4	50.0	42.589^a^	< 0.001	0.233^c^	< 0.001
Medium (3,001–6,000)	9.2	21.2				
High (6,001 ≥)	17.4	28.8				
Land assets						
Without land property	92.6	76.8	35.822^b^	< 0.001	0.222^d^	< 0.001
With land property	7.4	23.2				
***Livelihood assets***						
Human capital						
Low (≤ 12)	48.5	43.6	21.272^a^	< 0.001	0.165^c^	< 0.001
Medium (13–14)	37.2	28.4				
High (15 ≥)	14.3	28.0				
Social capital						
Low (≤ 7)	41.4	27.1	14.588^a^	< 0.001	0.137^c^	< 0.001
Medium (8–9)	37.4	47.9				
High (10 ≥)	21.2	25.0				
Natural capital						
Low (≤ 8)	67.0	45.8	51.382^a^	< 0.001	0.256^c^	< 0.001
Medium (9–10)	18.1	42.4				
High (11 ≥)	14.8	11.9				
Physical capital						
Low (≤ 9)	46.3	42.8	10.122^a^	0.006	0.114^c^	0.006
Medium (10–11)	41.0	50.8				
High (12 ≥)	12.6	6.4				
Financial capital						
Low (≤ 9)	44.0	21.6	36.360^a^	< 0.001	0.216^c^	< 0.001
Medium (10–11)	29.3	37.3				
High (12 ≥)	26.7	41.1				
Political capital						
Low (≤ 1)	64.8	43.2	56.504^a^	< 0.001	0.269^c^	< 0.001
Medium (2–3)	27.5	30.5				
High (4 ≥)	7.7	26.3				
***Household vulnerability***						
Social vulnerability						
Low (≤ 3.5)	37.7	35.6	0.420^a^	0.811	0.023^c^	0.811
Medium (3.51–4.25)	35.3	35.6				
High (4.26 ≥)	26.9	28.8				
Economic vulnerability						
Low (≤ 4.33)	35.3	47.0	10.489^a^	0.005	0.116^c^	0.005
Medium (4.34–5.67)	31.0	22.5				
High (5.68 ≥)	33.7	30.5				
Physical vulnerability						
Low (≤ 4.33)	31.0	44.1	21.193^a^	< 0.001	0.165^c^	< 0.001
Medium (4.34–5.67)	33.2	35.6				
High (5.68 ≥)	35.9	20.3				
Institutional vulnerability						
Low (≤ 6.50)	30.2	46.2	26.361^a^	< 0.001	0.184^c^	< 0.001
Medium (6.51–9.50)	35.0	35.2				
High (9.51 ≥)	34.8	18.6				
Attitudinal vulnerability						
Low (≤ 4.00)	30.8	47.9	21.076^a^	< 0.001	0.164^c^	< 0.001
Medium (4.01–5.00)	52.9	40.7				
High (5.01 ≥)	16.3	11.4				
Environmental vulnerability						
Low (≤ 1.50)	12.3	25.0	21.629^a^	< 0.001	0.166^c^	< 0.001
Medium (1.51–2.00)	31.9	31.8				
High (2.01 ≥)	55.9	43.2				
***Household food insecurity access***						
Food secure (≤ 1)	1.1	4.2	61.739^a^	< 0.001	0.281^c^	< 0.001
Mild food insecurity (2–7)	15.4	34.7				
Moderate food insecurity (8–14)	72.7	60.2				
Severe food insecurity (15 ≥)	10.8	0.8				

^**a**^ Pearson’s Chi-square (χ^2^); ^**b.**^ Yates’ continuity correction (χ^2^_Yates_); ^**c.**^ Cramer’s V (*φ*_c_); ^**d.**^ Phi (*φ*)

The findings from Table 1 further suggested that among socio-demographic characteristics of SMFRDCs, Muslims enjoyed better diversity than their Hindu counterparts (χ^2^_Yates_ [1, *n* = 782] = 3.715, *p* = 0.054; *φ* = 0.075), while SMFRDCs with secondary and higher education had better dietary diversity (χ^2^ [2, *n* = 782] = 21.945, *p* < 0.001; *φ*_c_ = 0.168). Likewise, SMFRDCs engaged in a single or three seasonal occupations seemingly enjoyed better dietary diversity (χ^2^ [3, *n* = 782] = 7.781, *p* = 0.051; *φ*_c_ = 0.100), whereas SMFRDCs from Shyamnagar and Koyra *Upazila* apparently had higher dietary diversity than those from Mongla *Upazila* (χ^2^ [2, *n* = 782] = 19.090, *p* < 0.001; *φ*_c_ = 0.156). Regarding family type, nuclear family members had higher dietary diversity than extended family members (χ^2^_Yates_ [1, *n* = 782] = 11.593, *p* < 0.001; *φ* = 0.125). Furthermore, the findings show that households with savings (χ^2^_Yates_ [1, *n* = 782] = 17.081, *p* < 0.001; *φ* = 0.151) and loans (χ^2^_Yates_ [1, *n* = 782] = 10.153, *p* < 0.001; *φ*_c_ = 0.117) enjoyed higher dietary diversity compared to those without savings and those with no loans.

Regarding household assets, the findings showed that SMFRDCs with better household construction materials and facilities (χ^2^ [2, *n* = 782] = 10.973, *p* = 0.004; *φ*_c_ = 0.118) and higher domestic assets (χ^2^ [2, *n* = 782] = 15.919, *p* < 0.001; *φ*_c_ = 0.143) enjoyed comparatively higher dietary diversity within their households than those with lower household facilities and domestic assets. Likewise, SMFRDCs with higher transport (χ^2^ [2, *n* = 782] = 16.120, *p* < 0.001; *φ*_c_ = 0.144) and livestock assets (χ^2^ [2, *n* = 782] = 42.589, *p* < 0.001; *φ*_c_ = 0.233) seemingly enjoyed a sharp rise of dietary diversity, whereas holding land assets (χ^2^_Yates_ [1, *n* = 782] = 35.822, *p* < 0.001; *φ* = 0.222) also boosted the dietary diversity among the SMFRDCs.

Among livelihood assets, four of six capitals contributed positively to higher dietary diversity among the SMFRDCs. The findings showed that SMFRDCs with higher human capital (χ^2^ [2, *n* = 782] = 21.272, *p* < 0.001; *φ*_c_ = 0.165), social capital (χ^2^ [2, *n* = 782] = 14.588, *p* < 0.001; *φ*_c_ = 0.137), financial capital (χ^2^ [2, *n* = 782] = 36.360, *p* < 0.001; *φ*_c_ = 0.216), and political capital (χ^2^ [2, *n* = 782] = 56.504, *p* < 0.001; *φ*_c_ = 0.269) enjoyed better dietary diversity than those with lower such capitals. In contrast, high natural capital (χ^2^ [2, *n* = 782] = 51.392, *p* < 0.001; *φ*_c_ = 0.256) and physical capital (χ^2^ [2, *n* = 782] = 10.122, *p* = 0.006; *φ*_c_ = 0.114) among SMFRDCs seemingly did not ensure higher dietary diversity within their households.

Regarding household vulnerability, it is apparent that households with lower vulnerability in any form, except social ones, contributed positively to better dietary diversity among the SMFRDCs. The findings showed that lower economic (χ^2^ [2, *n* = 782] = 10.489, *p* = 0.005; *φ*_c_ = 0.116), physical (χ^2^ [2, *n* = 782] = 21.193, *p* < 0.001; *φ*_c_ = 0.165), institutional (χ^2^ [2, *n* = 782] = 26.361, *p* < 0.001; *φ*_c_ = 0.184), attitudinal (χ^2^ [2, *n* = 782] = 21.076, *p* < 0.001; *φ*_c_ = 0.164), and environmental vulnerability (χ^2^ [2, *n* = 782] = 21.629, *p* < 0.001; *φ*_c_ = 0.166) increased the chances of better dietary diversity among the SMFRDCs in the SMF.

The study’s findings further showed that households with severe food insecurity among the SMFRDCs (χ^2^ [3, *n* = 782] = 61.739, *p* < 0.001; *φ*_c_ = 0.281) were more at risk of poorer dietary diversity.

### Determinants of household dietary diversity among SMFRDCs

Table 2 showed the determinants of dietary diversity among the SMFRDCs in southwestern coastal Bangladesh. The adjusted MBLR model, including all predictors, was statistically significant (χ2 [45, n = 782] = 303.362, p < 0.001), indicating that the model successfully distinguished between SMFRDCs with no or low dietary diversity and those with medium or high dietary diversity. Overall, the model explained between 33.1% (Cox & Snell R^2^) and 47.1% (Nagelkerke R^2^) of the variance in dietary diversity among SMFRDCs and correctly classified 82.8% of the cases.

**Table 2:** Determinants of dietary diversity among SMFRDCs.

Variables		B (SE)	*P* value	Exp(B)	95% CI	
					Lower	Upper
***Socio-demographic and economic characteristics***						
Religion						
Islam ^r^		1.000				
Hindu		0.168 (0.422)	0.691	1.183	0.517	2.703
Education						
Not literate ^r^		1.000				
Primary (Class I – Class V)		0.063 (0.252)	0.802	1.065	0.650	1.746
Secondary or higher (Class VI ≥)		1.058 (0.339)	**0.002**	2.880	1.481	5.598
*Upazila*						
Shyamnagar ^r^		1.000				
Koyra		−0.276 (0.292)	0.344	0.759	0.428	1.344
Mongla		−1.068 (0.299)	**< 0.001**	0.344	0.191	0.617
Type of family						
Nuclear		1.000				
Extended		−0.227 (0.272)	0.403	0.797	0.468	1.357
Savings						
No ^r^		1.000				
Yes		−0.063 (0.293)	0.830	0.939	0.529	1.667
Loans						
No ^r^		1.000				
Yes		0.097 (0.270)	0.720	1.101	0.649	1.869
***Transforming structures and processes***						
Seasonal occupation						
One ^r^		1.000				
Two		−0.202 (0.259)	0.434	0.817	0.492	1.356
Three		−0.157 (0.344)	0.648	0.855	0.436	1.677
Four		1.347 (0.681)	**0.048**	3.845	1.012	14.611
***Household assets***						
Household construction materials and facilities						
Low (≤ 18) ^r^		1.000				
Medium (19–20)		−0.294 (0.265)	0.267	0.745	0.443	1.253
High (21 ≥)		−0.017 (0.287)	0.952	0.983	0.560	1.726
Domestic assets						
Low (≤ 4,500) ^r^		1.000				
Medium (4,501−11,900)		0.929 (0.305)	**0.002**	2.532	1.393	4.601
High (11,901 ≥)		0.485 (0.304)	0.111	1.624	0.895	2.948
Transport assets						
Low (≤ 10,000) ^r^		1.000				
Medium (10,001–20,000)		0.640 (0.273)	**0.019**	1.896	1.111	3.238
High (20,001 ≥)		0.582 (0.328)	**0.076**	1.790	0.942	3.403
Livestock assets						
Low (≤ 3,000) ^r^		1.000				
Medium (3,001–6,000)		0.853 (0.342)	**0.013**	2.347	1.200	4.590
High (6,001 ≥)		0.495 (0.313)	0.114	1.641	0.888	3.030
Land assets						
Without land property ^r^		1.000				
With land property		0.344 (0.364)	0.345	1.411	0.691	2.880
***Livelihood assets***						
Human capital						
Low (≤ 12) ^r^		1.000				
Medium (13–14)		−0.587 (0.288)	**0.042**	0.556	0.316	0.978
High (15 ≥)		0.193 (0.377)	0.609	1.213	0.579	2.539
Social capital						
Low (≤ 7) ^r^		1.000				
Medium (8–9)		0.547 (0.272)	**0.044**	1.728	1.014	2.945
High (10 ≥)		−0.330 (0.362)	0.362	0.719	0.354	1.461
Natural capital						
Low (≤ 8) ^r^		1.000				
Medium (9–10)		1.105 (0.272)	**< 0.001**	3.021	1.772	5.150
High (11 ≥)		−0.416 (0.361)	0.249	0.660	0.325	1.337
Physical capital						
Low (≤ 9) ^r^		1.000				
Medium (10–11)		−0.222 (0.262)	0.396	0.801	0.480	1.338
High (12 ≥)		−1.518 (0.468)	**0.001**	0.219	0.088	0.549
Financial capital						
Low (≤ 9) ^r^		1.000				
Medium (10–11)		0.660 (0.286)	**0.021**	1.935	1.106	3.387
High (12 ≥)		0.354 (0.349)	0.310	1.425	0.719	2.822
Political capital						
Low (≤ 1) ^r^		1.000				
Medium (2–3)		0.338 (0.302)	0.262	1.402	0.776	2.532
High (4 ≥)		2.139 (0.371)	**< 0.001**	8.488	4.101	17.569
***Household vulnerability***						
Economic vulnerability						
Low (≤ 4.33) ^r^		1.000				
Medium (4.34–5.67)		−0.461 (0.290)	0.111	0.630	0.357	1.112
High (5.68 ≥)		−0.076 (0.297)	0.799	0.927	0.518	1.660
Physical vulnerability						
Low (≤ 4.33) ^r^		1.000				
Medium (4.34–5.67)		0.036 (0.275)	0.896	1.036	0.605	1.777
High (5.68 ≥)		−0.676 (0.307)	**0.027**	0.509	0.279	0.927
Institutional vulnerability						
Low (≤ 6.50) ^r^		1.000				
Medium (6.51–9.50)		−0.219 (0.330)	0.508	0.803	0.420	1.536
High (9.51 ≥)		−0.650 (0.380)	**0.087**	0.522	0.248	1.098
Attitudinal vulnerability						
Low (≤ 4.00) ^r^		1.000				
Medium (4.01–5.00)		−0.114 (0.270)	0.673	0.892	0.526	1.515
High (5.01 ≥)		−0.211 (0.402)	0.600	0.810	0.368	1.782
Environmental vulnerability						
Low (≤ 1.50) ^r^		1.000				
Medium (1.51–2.00)		0.354 (0.355)	0.318	1.425	0.711	2.858
High (2.01 ≥)		0.400 (0.383)	0.296	1.492	0.705	3.160
***Household food insecurity access***						
Food secure (≤ 1) ^r^		1.000				
Mild food insecurity (2–7)		0.137 (0.735)	0.852	1.147	0.272	4.843
Moderate food insecurity (8–14)		−0.543 (0.727)	0.454	0.581	0.140	2.413
Severe food insecurity (15 ≥)		−4.529 (1.287)	**< 0.001**	0.011	0.001	0.135
**Model Statistics**						
− 2 Log likelihood =		**614.063**				
Cox & Snell R^2^		**0.331**				
Nagelkerke R^2^		**0.471**				
**Hosmer-Lemeshow Test**		**χ** ^**2**^**= 10.746, df = 8, *p* > 0.271**				
**Classification**						
*% correct*	No/Low dietary diversity (≤ 5) -	**94.5%**				
	Medium/High dietary diversity (6 ≥)	**54.9%**				
	Overall	**82.8%**				

B. Unstandardized regression weight; ^**SE.**^ Standard error; ^**Exp (B).**^ Predicted change in odds for an increase in the predictor(s);

CI . Confidence interval; ^**df.**^ Degrees of freedom; ^**ref.**^ Reference category.

The findings showed that variables related to transforming structures and processes, as well as livelihood strategies, have mixed effects on the dietary diversity of SMFRDCs. For example, SMFRDCs with secondary and higher education were 2.880 times more likely to have a higher dietary diversity. Likewise, SMFRDCs engaged in four different seasonal occupations were 3.845 times more likely to have better dietary diversity. In contrast, SMFRDCs in Mongla *Upazila* were 0.344 times less likely to have higher dietary diversity than those in Shyamnagar *Upazila*.

The findings indicated that household assets, more specifically moderate access to domestic, transport, and livestock, positively influenced the dietary diversity of SMFRDCs. It was found that SMFRDCs with moderate domestic assets were 2.532 times more likely to have a better dietary diversity than those with lower domestic assets. Likewise, households with medium to high transport assets were 1.896 and 1.790 times more likely to have higher dietary diversity than households with lower transport assets, respectively. In a similar vein, SMFRDCs with medium livestock assets were 2.347 times more likely to have better dietary diversity than households with lower livestock assets.

Regarding livelihood assets, they were found to have mixed impacts on the dietary diversity of SMFRDCs in the SMF. For example, households with medium human capital and high physical capital were 0.556 and 0.801 times less likely to have better dietary diversity, respectively. In contrast, SMFRDCs with medium levels of social, natural, and financial capital were 1.728, 3.021, and 1.935 times more likely to have higher household dietary diversity, respectively. Similarly, households with high political capital were 8.488 times more likely to secure better dietary diversity for their family members.

The findings showed that household vulnerabilities were negatively affecting the dietary diversity of SMFRDCs. For example, SMFRDCs with high physical vulnerability were 0.509 times less likely to have a diversified family diet. At the same time, households with high institutional vulnerability were 0.522 times less likely to have dietary diversity.

Regarding household food insecurity, it is evident that SMFRDCs with severe food insecurity were 0.011 times as likely to have a diversified diet as households with no food insecurity.

## Discussion

This study, based on the Sustainable Livelihood Approach (SLA), assessed the prevalence of dietary diversity among SMFRDCs in southwestern coastal Bangladesh. Overall, less than 30% of forest resource-dependent households have diversified diets, and dietary diversity was more prevalent among honey collectors and households in Shyamnagar *Upazila*. It was found that certain elements of SLA framework proved to be critical to understand the dynamics of dietary diversity. Using multivariable binary logistic regression, household assets and livelihood assets were the most important determinants of dietary diversity among forest-proximate households. In addition, sociodemographic factors, such as the household head’s education and household spatial location, as well as transforming factors, including seasonal occupations, were important predictors of dietary diversity among SMFRDCs. The significance of household vulnerability and food insecurity in predicting dietary diversity proved irrefutable in this study; however, livelihood strategies were not found to be a deciding factor for SMFRDCs.

### Prevalence of dietary diversity

The findings of the current study show that just over a quarter of SMFRDCs’ households have a diversified diet. Among the SMFRDCs, honey collectors (34.3%) and those living in Shyamnagar *Upazila* (37.1%) had higher dietary diversity than other households in the Sundarbans. The higher prevalence of poor dietary diversity among SMFRDCs can be attributed to seasonal unemployment, when these forest-dependent people were barred from entering the Sundarbans for resource extraction [23]. In addition, the diminishing demand for forest products, such as *Nypa* leaf as a thatching material, may also have played an important role [6]. However, it cannot be denied that certain high-end non-perishable forest products, including honey and wax, have a better financial return [24] because of its commercial value as well as promotion through media as a profitable product [87], and concentration of particular occupational groups to specific regions, such as honey collectors in Shyamnagar *Upazila* of Satkhira district [88] may have led to better dietary diversity among SMFRDCs in certain occupations and spatial locations. Unlike the SMFRDCs, it is evident that rice producers in Bangladesh had greater dietary diversity, with a negligible percentage (0.11%) having low dietary diversity, while 16.2% and 83.7% had medium and high dietary diversity, respectively [79]. In Sylhet and Moulvibazar, the average HDDS was 7.16 [19], higher than the national average [13,14], and 64.5% of households had high dietary diversity [19]. Likewise, Kundu, Banna (11) noted that more than 85% of households across Bangladesh had high (41.5%) or moderate (44.9%) HDDS during the COVID-19 pandemic. It is also evident that dietary diversity may vary within an occupational group depending on spatial location, as a study on tea workers indicated that 65% and 50.5% of tea-working households in two selected tea gardens had medium to high dietary diversity [80]. In contrast, Tasnim and Karim [89] found that nearly three out of five women (59.9%) had low dietary diversity, and the rest (40.1%) had moderate to high dietary diversity during the COVID-19 pandemic in Bangladesh. Likewise, a study on animal herders in Zimbabwe showed that more than 55% of households have a low dietary diversity, reflecting more profound food insecurity among rural animal herders [90]. These findings indicate that, even during crisis periods, non-forest dependents enjoyed greater dietary diversity than SMFRDCs under normal circumstances.

### Socio-demographic, economic characteristics, and dietary diversity

The findings showed that education played a decisive role in ensuring dietary diversity among forest-dependent households, where SMFRDCs with secondary or higher education had a more diverse diet than those with primary or no education. It is well documented that educated people can diversify their livelihood options under any circumstances; therefore, breaking the poverty cycle, which could potentially lead to higher income and better ability to provide necessary food for their household members [20,80]. Studies further suggest that households with an educated mother or an educated male household head could play a decisive role over non-literate or female-headed households due to higher purchasing power, better nutritional knowledge and planning, preparation of suitable and affordable recipes for family members, as well as appropriate cooking practices to improve dietary diversity [13,20,91]. As in the current findings, Kundu et al. (2021) also observed that higher levels of education among household heads were significantly and positively associated with greater dietary diversity among household members during the COVID-19 pandemic. Likewise, Alamirew, Lemke (92) found that people with higher levels of education were more likely to have better access to nutritious food than those with no formal education. A recent study on forest resource-dependent people also corroborates the current study’s findings, suggesting that higher education among household heads not only ensures greater dietary diversity but also greater access to animal protein for household members [18].

The findings indicated that households in Mongla *Upazila* were less likely to have a diversified diet than those in Shyamnagar *Upazila*. This implies that SMFRDCs in Mongla have the least dietary diversity, which can be attributed to exposure to frequent natural disasters [10], that may have negatively affected the household’s socio-economic conditions, including ownership of assets or wealth [26], thus leading to food insecurity during crisis moments [92], especially during seasonal unemployment or other similar emergency situations [8,9]. Additionally, reduced purchasing power, along with inappropriate cooking practices and recipe choices, cannot be ruled out as possible reasons for poor dietary diversity among households in Mongla *Upazila* [13]. A similar result was documented among tea workers by Ahmed, Mozahid (80) who observed that tea workers in Sylhet *Sadar* (central) *Upazila* had higher dietary diversity than tea workers in Rajnagar *Upazila*, as the tea workers in *Sadar Upazila* had lower family size and dependency, and were more involved in diversified occupations due to greater literacy rates, which eventually led to better food security and dietary diversity through higher income in secured employment opportunities [11,80]. Likewise, Hossain, Shohel (18) found that some forest resource-dependent communities may have an advantage over others due to a high concentration of valuable and non-perishable forest resources, such as honey and wax [88] that not only ensured higher household income but also secured access to nutritious, affordable diets necessary for the physical well-being of their family members.

### Transforming structures, processes, and dietary diversity

It was also evident that engaging in multiple seasonal occupations significantly increased the likelihood of dietary diversity among SMFRDCs in Sundarbans. It is well documented that Sundarbans-proximate households often get involved in secondary occupations under normal circumstances [6,93] as well as during crisis periods, such as the COVID-19 pandemic [8,9], natural disasters [10,49], and during moratorium – a ban imposed by the Forest Department to prevent resource extraction from the Sundarbans [23], to earn additional household income to meet daily necessities, and to ensure the overall wellbeing of the households [6,8,9,93], including access to nutritious food items [18]. Otherwise, due to the absence or unreliability of income sources, especially during crisis periods, marginalized people were forced to migrate to cities in search of alternative livelihoods [68], or they were compelled to borrow money either from relatives without paying any interest or, in most cases, from local moneylenders (locally known as *Mahajan*) with high interest; thus straining the forest resource-dependent households to be burdened with loan repayments [6,8,9,23,49], which may shrink their purchasing capacity or affordability of necessary food items for consumption [22]. Similar to the findings of this study, Akter, Yagi (91) concluded that taking on multiple jobs enabled poor households to generate substantial income to purchase necessary nutritional food items, thereby ensuring a relatively better-quality diet for their family members than households with a single underpaid job. Likewise, a previous study on dietary diversity during the COVID-19 pandemic found that insecure employment was strongly associated with lower dietary diversity among people in Bangladesh [11]. A recent study also suggested that additional income from multiple jobs enabled forest-proximate households to access a diversified range of food items, mostly plant-based, because animal-based foods were beyond their means [18].

### Household assets and dietary diversity

It was found that households’ domestic assets significantly and positively influenced dietary diversity among the SMFRDCs. Research suggests that higher domestic assets serve as indicators of a household’s wealth, which inadvertently empowers the household heads to secure food items during crises [92]. Households with higher domestic assets were less likely to spend cash on goods, such as household furniture; rather, they could mobilize more resources to buy food for their household members, thereby ensuring higher dietary diversity [18,92], whereas households with the least domestic assets were evidently experiencing lower dietary diversity [26], due to difficulties in purchasing food items [22]. A similar result was documented among rural women in Zambia, where it was evident that domestic assets or wealth positively and significantly influenced dietary diversity because households with better domestic assets were more likely to spend money on food items; therefore, they had more dietary diversity [87].

Similarly, transport assets also contributed positively to dietary diversity among forest-proximate households, indicating that households with transport facilities were more likely to have better dietary diversity. Households with access to private vehicles were more likely to have a higher dietary diversity than households without private vehicles [94]. Because people living in isolated areas without private vehicles were less interested in commuting or walking for a distance longer than 4 hours; hence, they have a limited choice of food items due to limited access to marketplaces to buy nutritious food items, increasing the risk of lower dietary diversity [94,95] and loss of body mass index [96]. In remote mountainous areas, having livestock, such as horses or cows, in households is considered a blessing that allowed people to transport necessary items, such as staple foods, rice, chicken, and other plant products, from distant marketplaces, thus ensuring better food security as well as a diversified diet [94,97]. A recent study on forest resource-dependent communities in the Sundarbans found that access to transport positively contributed to dietary diversity, especially plant-based foods [18].

Likewise, livestock assets significantly influenced households’ dietary diversity among forest-proximate households, indicating that households with livestock assets were more likely to experience higher dietary diversity. Ownership of livestock, including cattle, goats, or sheep, and poultry, could enable households to slaughter them as a source of nutrient-rich foods for their family members or sell them as valuable resources when needed to sustain their livelihoods or to purchase necessary groceries for households, especially during emergencies [8,9,98]. The literature suggested that livestock ownership significantly and positively determined the dietary diversity of rural households in Bangladesh, including among forest resource-dependent communities [18,19]. Similarly, Usman and Callo-Concha (100) found that households with livestock had higher and more diverse diets than households without livestock. However, Manyeruke et al. (2023) noted that the absence of livestock in a household negatively but not significantly affected dietary diversity among animal herders in rural Zimbabwe.

### Livelihood assets and dietary diversity

The findings showed that households’ human capital was inversely associated with dietary diversity, indicating that households with more members depending on natural resources for livelihood were less likely to have a more diversified diet. It is well documented that the southwestern coastal *Upazilas* have larger families, including children and aged people, than the national household size [99–101]. Thus, it is imperative that these households may experience lower dietary diversity due to greater dependency or the absence of actively earning members to purchase a range of diversified food items [11,19]. Furthermore, the lack of education and employment opportunities among household members, especially household heads during natural and man-made disasters, the work overload on earning members, along with a lack of support in household chores and in decision-making, may also lead households to poor dietary diversity [11,18,102]. Studies in Zambia and China also showed that families with a child with a disability or a member with obesity had a higher likelihood of lower dietary diversity [103,104]. In addition, the household head’s involvement in lower-income generating activities (IGAs) reduced households’ capacity to buy protein-enriched food items, such as eggs, fish, chicken, and meat; thus, household managers, for example, women, relied on low-nutrient food items available within the reach of households, that is, vegetables, to feed the household members [11,21]. In contrast, Zhang, Zhang (106) and Zhang, Chang (105) found that household members’ involvement in non-farm employment was positively and significantly associated with increased intake of animal- and plant-based protein, as well as higher dietary diversity due to higher income and better awareness regarding diversified diets through exposure to the internet. Moreover, family size, family type, age, and educational qualifications of the household head and its members also determined dietary diversity [11,15,105].

Among livelihood assets, social capital was positively associated with dietary diversity among SMFRDCs, suggesting that social connectedness within communities was more likely to enhance dietary diversity among households in the Sundarbans-proximate southwestern coastal regions. Marginalized communities, such as SMFRDCs, often sought help, either financial or in-kind, from their relatives and neighbors during difficult periods to address unforeseen events or unanticipated circumstances, such as seasonal unemployment or natural disasters, through mutual trust and benefits [8,9,49]. A similar result was documented in a recent study by Hossain, Shohel (18) among forest resource-dependent communities in the Sundarbans, who observed that social capital not only increased overall dietary diversity but also enhanced the likelihood of consuming animal-based proteins among these marginalized communities. Likewise, Alam, Begum (15) concluded that people’s social capital, as reflected in market participation and access to information through an integrated community, positively influenced the dietary diversity of men, women, and children in Bangladesh. Alamirew, Lemke (92), on the contrary, observed that certain forms of social capital, i.e., sociocultural beliefs regarding gender, undermined women’s status within and outside their households, discouraging them from consuming a diverse range of food items and, in turn, reducing their access to adequate dietary diversity.

It was found that the natural capital of SMFRDCs was positively associated with dietary diversity, indicating that households with higher levels of natural capital were more likely to experience a diverse range of food items. The forest-proximate communities extracted resources from the Sundarbans and adjacent areas, and delivered them to nearby marketplaces, either directly or through the local moneylenders they were indebted to [6,8,9,49,93,106], to get a quick monetary return that may have allowed them to buy necessary groceries, including food items, for their households, thus increasing dietary diversity within their families [18,93]. Moreover, living closer to rivers may also give them an advantage in accessing animal protein from fish [23]. In a similar vein, it is documented that a favorable climate, along with access to irrigated land, significantly increased dietary diversity among households in the mountainous regions of Afghanistan [95]. In contrast, it is evident that certain natural capital, such as higher temperatures or greater distance to the marketplace, negatively affected the agricultural production of coastal communities, thereby limiting their dietary diversity [21,106].

Like human capital, this study found that physical capital was negatively associated with dietary diversity, meaning that households with the highest levels of physical capital were more likely to experience lower dietary diversity. Households with better physical capital may experience minimal dietary diversity due to inadequate institutional support, including limited market engagement and connectivity [87], as well as the low purchasing power of socioeconomically and environmentally vulnerable communities [14]. Besides, being overly dependent on natural resources for livelihood, which is often affected by climate variability, might have also reduced dietary diversity among marginalized people [107] Alamirew, Lemke (92), on the contrary, found that physical capital, that is, access to potable water, significantly and positively influenced reproductive-aged women’s access to a proper and nutritious diet.

The findings of this study suggested that households with greater financial capital were more likely to have higher dietary diversity. Although the forest-proximate households in the Sundarbans did not make much from resource extraction [6,23], especially during a moratorium or similar other restrictions [8,9,23], the abundance of edible vegetation, along with fisheries items, including shrimp, crab, and fish, and the lower price of necessary food items, may have allowed these marginalized communities to have access to a range of food items needed for their households, and thus enjoyed a diversified diet. Likewise, Kundu, Banna (11) and Weerasekara, Withanachchi (108) noted that higher-income families were more likely to enjoy greater dietary diversity than lower-income families, as they had more resources and financial capabilities to make necessary food items available. Similarly, Zhang, Chang (105) observed that households with higher incomes in China have greater dietary diversity than those with lower incomes. It is important to note that dietary diversity is a complex issue in which the price and availability of desired products guide households’ choices, and lower-income families, owing to financial constraints, were unable to spend more money on the required or desired food items [87,108]. However, producing goods, such as food items – whether vegetables or livestock and fish, in their own farms, households may have better dietary diversity, irrespective of their economic conditions [106,109].

Similar to financial capital, political capital was positively associated with dietary diversity among the SMFRDCs. The findings indicate that households with high political capital contributed positively and significantly to moderate to better dietary diversity. There is no denial that forest-proximate households in the Sundarbans go through a phase of involuntary unemployment every year due to seasonal variability of forest products, such as honey and wax, as well as restrictions imposed by the Forest Department in order to ensure sustainable resource extraction by reducing biodiversity loss through protecting breeding season and revival of forest health, such as *Nypa* leaf and fish fry [6,23,49,110]. However, they were compensated in the form of cash payments or food aid [111,112], which was often mismanaged by local political leaders. Without political connections, SMFRDCs do not receive such compensation during seasonal unemployment. Thus, involvement in political activities or connections with local political leaders has been a crucial issue for SMFRDCs in accessing the government’s support to ensure food security and dietary diversity [18]. Similar results were reported by Zhang, Zhang (106), who observed that individuals with a strong presence in rural communities in China increased their likelihood of consuming animal- and plant-based protein, as well as their dietary diversity.

### Household vulnerability and dietary diversity

Regarding household vulnerability, it was found that physical vulnerability negatively affected dietary diversity among SMFRDCs, indicating that households with low physical capacity, especially during climate-induced natural disasters, had lower dietary diversity. A recent study in southwestern coastal Bangladesh found that households with low or no physical capacity to cope with emerging vulnerabilities experienced crop losses, followed by reduced food consumption and poor dietary diversity [25]. Similarly, Rahman, Chowdhury [113] and Hidalgo, Witten (21) argued that climatic events affect not only the lives and livestock but also damage the physical infrastructure and properties of coastal people, which inadvertently reduces the household’s capacity to recover from the vulnerable situation due to reduced income, thereby leading to lower food consumption and poor dietary diversity. Likewise, Hossain, Shohel (18) found that the lack of institutional support during and post-disaster situations, especially among forest-proximate communities, reduced dietary diversity, including both plant- and animal-based diets.

### Household food insecurity and dietary diversity

Regarding food insecurity and dietary diversity, the findings show that households with severe food insecurity were more likely to have lower dietary diversity, suggesting that severe food insecurity was strongly associated with low or no dietary diversity among the SMFRDCs. The forest-proximate households, due to seasonal variability and protective measures to revive wildlife and forest health [6,23,110], often experience an involuntary unemployment that may have not only reduced their overall income [6,93], but also led to severe food insecurity; therefore, it shrank dietary diversity [18]. A similar result was reported by Ali, Raihan (19), who found that households with severe food insecurity were more likely to experience lower dietary diversity. Similarly, Kundu, Banna (11) observed a strong association between poor food security and lower dietary quality among the general population during the COVID-19 pandemic in Bangladesh. Ahmed, Mozahid (80) also found a strong association between food security and dietary diversity among tea workers, and reported that higher calorie intake was significantly and positively linked with better food security and dietary diversity. On the contrary, a study on adolescents in Nigeria found no significant link between food insecurity and dietary diversity, yet the authors acknowledged the possibility of an indirect relationship [114].

## Strengths and limitations

This study had several strengths. To the best of our knowledge, this study is the first to examine the prevalence and determinants of dietary diversity among SMFRDCs, a marginalized forest-dependent population in southwestern coastal Bangladesh, to identify their challenges with healthy diets and the associated impediments. Administering a structured interview schedule (SIS) in face-to-face home settings enabled the researchers to obtain not only reliable information but also a reflexive understanding of the informant’s experience and perceptions regarding dietary diversity. The use of a rigorous sampling process in the investigation, i.e., multi-stage stratified random sampling, allowed the researchers to cover substantial geographical areas with varied participant experiences through unbiased selection, thereby ensuring the generalizability of the findings without prejudice. Moreover, the use of universally validated and reliable scientific tools to measure dietary diversity, along with relevant variables based on a comprehensive theoretical framework, with maximum and minimum values for a range of sub-components, and strict quality control to obtain high-quality, reliable data, can be replicated in future research. Apart from these strongholds, readers must note some limitations as well. Relying on a quantitative approach to complex issues, such as the dietary diversity of a marginalized occupational group subject to frequent natural disasters, can yield numerical data without the holistic understanding that a mixed-methods approach enables. The cross-sectional design allowed the researcher to describe the association between variables without determining causality or direction. The estimation of dietary diversity was based on self-reported information from the past 24 hours using the HDDS; thus, recall bias may lead to over- or underestimation of food intake, despite the interviews being conducted by a group of well-trained data enumerators. The use of SIS may also lead to response bias among both informants and data enumerators due to socially desirable answers. Thus, longitudinal studies based on a mixed-methods approach should be designed to understand the complex dynamics of dietary diversity and temporal aspects along with the sociodemographic, economic, and politico-cultural issues of SMFRDCs in Bangladesh or FRDCs elsewhere.

## Conclusions and recommendations

**Fig 5:**
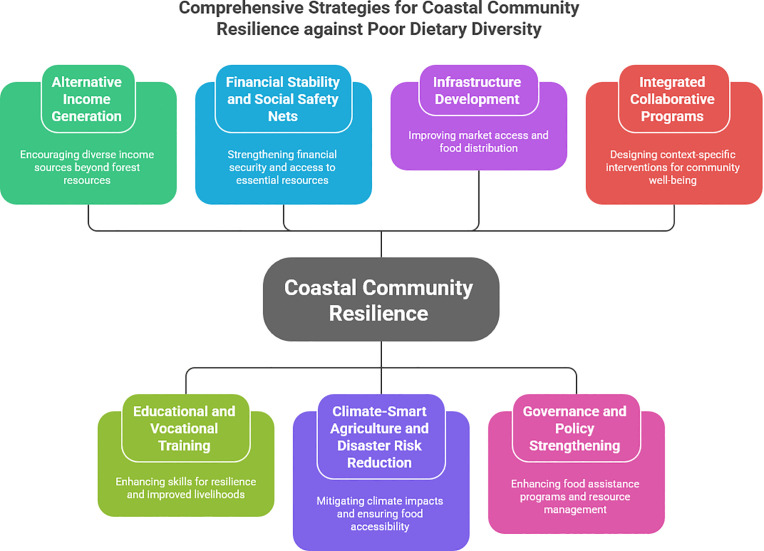
Policy recommendations to improve dietary diversity among SMFRDCs [115].

This study highlights the critical role of the sustainable livelihood approach, including household and livelihood assets, in shaping dietary diversity among Sundarbans mangrove forest resource-dependent communities (SMFRDCs) in southwestern coastal Bangladesh. The findings show that honey collectors and residents of Shyamnagar *Upazila* had higher dietary diversity. Similarly, household assets (domestic, transport, and livestock) and livelihood assets (human, social, natural, physical, financial, and political) significantly influenced dietary diversity of Sundarbans-proximate households. The education of household heads and their engagement in multiple seasonal occupations also positively impacted dietary diversity. It has also been documented that physical vulnerability and food insecurity significantly reduced dietary diversity among the SMFRDCs. Policymakers should prioritize the following measures (see Fig 5) to enhance dietary diversity among SMFRDCs – the most marginalized population in southwestern coastal Bangladesh: (i) encouraging SMFRDCs and their family members to engage in alternative income-generating activities (AIGAs), such as aquaculture, ecotourism, handicrafts and products – molasses and juice from *Nypa* palm, rather than relying entirely on forest resource extraction to reduce dependence on the Sundarbans mangrove forest (SMF); (ii) strengthening institutional microfinance opportunities and establishing occupational cooperatives and social safety nets to enhance financial stability while protecting these households from the debt of local moneylenders and ensure access to food items necessary for both physical and mental well-being of household members; (iii) implementing targeted educational and vocational training programs tailored specifically for the SMFRDCs to enhance their livelihood resilience, especially during emergencies, to improve the overall dietary diversity by increasing awareness regarding the consumption of locally available nutritious foods; (iv) introducing climate-smart agriculture, sustainable fisheries, and disaster risk reduction programs, designed specifically for the coastal population, to mitigate the impact of natural hazards on food accessibility during emergencies; (v) investing in the development of infrastructure, i.e., roads, transportation, and connectivity, to facilitate the promotion and marketing of local foods, including both perishable and non-perishable food items, and to provide access to diverse food sources from outside through incentives; (vi) strengthening governance structures to improve access to food assistance programs, especially during emergencies and off-season, and promote policies that ensure the sustainable use of forest resources from the SMF without posing a threat to its biodiversity and ecosystem; and (vi) integrating empirical evidence-based collaborative programs and policies between government and non-government development organizations with active participation of local stakeholders, including social, economic, and political entities and communities, to design context-specific interventions aimed at improving not only dietary diversity and food security but also the overall physio-psychological and socioeconomical well-being of the coastal population, while protecting biodiversity of the SMF. By addressing these key areas, policymakers and development practitioners can help create a more sustainable livelihood environment for SMFRDCs, ensuring their long-term food security and well-being.

## Supporting information

S1 File Inclusivity-in-global-research-questionnaire. (DOCX)
